# Ginsenoside Re Mitigates Photooxidative Stress-Mediated Photoreceptor Degeneration and Retinal Inflammation

**DOI:** 10.1007/s11481-023-10073-y

**Published:** 2023-06-16

**Authors:** Jie Chang, Yujue Wang, Jing Xu, Xiaoye Du, Jingang Cui, Teng Zhang, Yu Chen

**Affiliations:** 1 Yueyang Hospital of Integrated Traditional Chinese and Western Medicine, Shanghai University of Traditional Chinese Medicine, Shanghai, 200437 China; 2 Clinical Research Institute of Integrative Medicine, Shanghai Academy of Traditional Chinese Medicine, Shanghai, 200437 China; 3 Laboratory of Clinical and Molecular Pharmacology, Yueyang Hospital of Integrated Traditional Chinese and Western Medicine, Shanghai University of Traditional Chinese Medicine, Shanghai, 200437 China

**Keywords:** Retinopathy, Photooxidative stress, Photoreceptor degeneration, Neuroinflammation, Ginsenoside Re

## Abstract

Loss of photoreceptors is the central pathology accountable for irreversible vision impairment in patients with photoreceptor degenerative disorders. Currently, mechanisms-based pharmacological therapies protecting photoreceptors from degenerative progression remain clinically unavailable. Photooxidative stress plays a pivotal role in initiating the degenerative cascade in photoreceptors. Meanwhile, photoreceptor degeneration interacts closely with neurotoxic inflammatory responses primarily mediated by aberrantly activated microglia in the retina. Thus, therapies with anti-oxidant and anti-inflammatory properties have been actively investigated for their pharmacological value in controlling photoreceptor degeneration. In the current study, we examined the pharmacological potentials of ginsenoside Re (Re), a naturally occurring antioxidant with anti-inflammatory activities, in photooxidative stress-mediated photoreceptor degeneration. Our results demonstrate that Re attenuates photooxidative stress and associated lipid peroxidation in the retina. Furthermore, Re treatment preserves the morphological and functional integrity of the retina, counteracts photooxidative stress-induced perturbation of the retinal gene expression profiles and mitigates photoreceptor degeneration-associated neuroinflammatory responses and microglia activation in the retina. Lastly, Re partially antagonizes the deleterious effects of photooxidative stress on müller cells, verifying its beneficial impact on retina homeostasis. In conclusion, the work here provides experimental evidence supporting novel pharmacological implications of Re in attenuating photooxidative stress-mediated photoreceptor degeneration and ensuing neuroinflammation.

**Supplementary Information:**

The online version contains supplementary material available at 10.1007/s11481-023-10073-y.

## Introduction

As an integral part of the central nervous system, the retina carries out an elegant function of vision formation that begins with the first-order sensory neuron, the photoreceptors. Thus, the morphological and functional integrity of photoreceptors is indispensable for normal vision. Photoreceptor degeneration is the primary pathological cause for progressive and irreversible vision impairment and vision loss in conditions ranging from the commonly encountered multifactorial age-related macular degeneration to the rare inherited disorders such as retinitis pigmentosa (Wright et al. 2010). At present, photoreceptor protective drug therapies are clinically unavailable. Active research effort is still needed to develop mechanisms-based treatments to prevent and/or delay the progression of photoreceptor degeneration.

Photoreceptors are naturally vulnerable to oxidative stress-induced damages, particularly, lipid peroxidation due to their unique anatomical, structural, metabolic and functional characteristics. Meanwhile, photoreceptors are under tremendous pressure of photooxidative stress owing to their innate light-sensing functions (Beatty et al. 2000; Remé 2005; Hunter et al. 2012). Thus, photoreceptor degeneration is universally underpinned by excessive photooxidative stress regardless of genetic or environmental etiologies (B. Domènech and Marfany 2020). In addition, photoreceptor degeneration is not only characterized by loss of photoreceptors per se, it is often exacerbated by exaggerated neuroinflammatory responses orchestrated by activated microglia in the retina. Photoreceptor degeneration can be caused by multiple mechanisms, for instance, genetic mutations, environmental insults or combination of both. However, microglial activation and neuroinflammatory responses are invariably elicited even by subtle changes in the retinal microenvironment due to photoreceptor degeneration (Karlstetter et al. 2010; Silverman and Wong 2018; Guadagni et al. 2019; Rashid et al. 2019; Rathnasamy et al. 2019). Antioxidants with the properties to counteract photooxidative stress and lipid peroxidation as well as are capable of suppressing microglial inflammatory activation hold potentials for protecting against the development and progression of debilitating photoreceptor degeneration.

The contribution of natural products, rich in antioxidants with anti-inflammatory activities, to the development of novel drug therapies has gained increased research attention (Atanasov et al. 2021). Meanwhile, supported by thousands of years of clinical practice, traditional Chinese medicine (TCM) constitutes a unique repertoire for the development of naturally occurring photoreceptor protective therapies. *Panax ginseng* is one such candidate. Implied by the name *panax*, meaning all healing, *panax ginseng* is equipped with a broad range of highly valued therapeutic effects that are in part attributed to the antioxidant and anti-inflammatory effects of its major chemical components, ginsenosides (Attele et al. 1999). Interestingly, the vision-enhancing activities of ginseng have also been documented in *Shennong’s Herbal*, the earliest pharmacopoeia in Chinese history from approximately 2000 years ago. Ginsenoside Re (Re), the most abundant ginsenoside found in *panax* ginseng, is a membrane active substance with well-established antioxidant ability to attenuate lipid peroxidation (Verstraeten et al. 2020). The neuroprotective effects of Re against ischemia/reperfusion-induced brain injuries are in part ascribed to the antioxidant activity of Re in suppressing lipid peroxidation (Zhou et al. 2006). Meanwhile, it has also been reported that Re is effective at suppressing the inflammatory responses in activated microglia (Madhi et al. 2021). However, it remains to be determined if Re is pharmacologically active in attenuating photooxidative stress-mediated photoreceptor degeneration and associated neuroinflammatory responses. The current study was thus performed to further address the pharmacological implications of the antioxidant and anti-inflammatory activities of Re in photooxidative stress-mediated photoreceptor degeneration.

## Methods

### Animals and Treatments

Four-week-old female Balb/c mice were purchased from Shanghai Laboratory Animal Research Center (Shanghai, China) and maintained under regulated laboratory conditions with a 12/12 h light-dark cycle and a controlled temperature set at 20 ± 2 °C. After 2-week routine acclimation, the mice dark-adapted for 24 h were subjected to the white light exposure delivered at 15,000 lx (LED Floodlight, 30 W, PHILIP) for 30 min. Re (purity > 98%, Cat. No. B21055, Shanghai Yuanye Biological Technology Co. Ltd, China) was dissolved in 30% DMSO. The light-exposed mice were treated with either 30% DMSO (vehicle) or the indicated doses of Re through intraperitoneal injection 15 min before the light exposure. The dark-adapted mice unexposed to the experimental light exposure were treated with the vehicle in the same manner to serve as the normal controls. The laboratory animal handling protocol was reviewed and approved by the Institutional Animal Care and Use Committee at Yueyang Hospital of Integrated Traditional Chinese and Western Medicine, Shanghai University of TCM (YYLAC-2019-021-1) and carried out in accordance with the recommendations of the NIH Guide for the Care and Use of Laboratory Animals.

### ***In situ*** Detection of ROS Production in the Retina

In situ detection of ROS production was performed as previously described (Chen et al. 2012; Bian et al. 2016). In brief, 22 h after the experimental light exposure, dihydroethidium (DHE) (Sigma-Aldrich, USA), a fluorescent probe specific for superoxide and hydrogen peroxide, was intraperitoneally injected at the dose of 20 mg/kg bw. Eye cups were made 2 h after DHE administration, fixed in 4% paraformaldehyde for 30 min and processed for cryosectioning to make 12 μm-thick cryosections. After nuclear staining by 4′,6-diamidino-2-phenylindole (DAPI) (D8200, ROCHE, USA), the sections were directly imaged to assess the red fluorescence of the oxidized DHE using a fluorescent microscope (DM6000B, Leica, Germany). For each section, at least 4 images spanning the superior, inferior and central retina were acquired using Leica LAS AF software with the same camera gain and exposure setup for all treatment groups. ImageJ was used to quantify the red fluorescent signals of the oxidized DHE in each image (image size: 322.22 × 240.74 μm). In brief, after selecting color and split channels, mean gray value and limit to threshold were checked under “set measurements”. Polygon tools were used to determine the area to be analyzed. Under “analyze”, the mean gray value was measured. The sections were from the similar planes of the retina. The images subjected to ImageJ analysis were from the same region of the retina and the target area selection was kept consistent across different experimental groups. The numbers from 4 images per section were averaged to generate the final number for each individual retina.

### Optical Coherence Tomography (OCT)

Image-guided OCT (OCT 2 with Micron IV, Phoenix Research labs, USA) was employed to obtain the full-retinal cross-sectional scans 7 d after the experimental light exposure as previously described (Wu et al. 2020, 2022). In brief, intraperitoneal injection of ketamine hydrochloride (82.5 mg/kg bw) and xylazine (8.25 mg/kg bw) was administered to induce anesthesia in the mice, followed by dilation of pupils using 1% tropicamide and OCT imaging. Five scans from the superior, central and inferior retinas were acquired and averaged automatically by the Phoenix Reveal OCT software (Phoenix Research labs, USA). The averaged scans were subjected to morphological evaluation of the retina. The thickness of the photoreceptor outer nuclear layer (ONL), inner segment (IS) and outer segment (OS) as well as the inner nuclear layer (INL) was measured using Insight Image Segmentation Software for the Phoenix OCT and Retinal Imaging System (Version 2.0.5490, Voxeleron LLC, USA).

### Electroretinography (ERG)

Seven days after the experimental light exposure, the mice were dark-adapted for 16 h and subjected to ERG analysis. In brief, the mice were anesthetized with a cocktail of ketamine hydrochloride (82.5 mg/kg bw) and xylazine (8.25 mg/kg bw). After 1% tropicamide-induced dilation of the pupils, ERG recording was proceeded under safe-light conditions (5 lx) using the universal testing and electrophysiological Ganzfeld system (Phoenix Research labs, USA) following the protocols as previously described (Wu et al. 2020; Wu et al. 2022). In brief, green light flashes (504 nm) were delivered at the intensity of -2 (0.5 msec duration and 5 s inter-stimulus-interval), -0.8 (1 msec duration and 5 s inter-stimulus-interval), 0.4 (1 msec duration and 10 s inter-stimulus-interval), 1.6 (1 msec duration and 20 s inter-stimulus-interval) and 3.1 (1 msec duration and 60 s inter-stimulus-interval) log cd•s•m^− 2^. Scotopic ERG responses were then analyzed using the LabScribe software (Phoenix Research Labs, USA).

### Gross Retinal Histology and Immunohistochemistry (IHC)

To examine the gross retinal morphology, eyeballs were enucleated 7 d after the experimental light exposure, fixed in 4% paraformaldehyde for 24 h and processed for paraffin sectioning. Four µm-thick paraffin sections were stained with hematoxylin and eosin (HE). Paraffin sections were also subjected to IHC examinations to probe the expression pattern of 4-hydroxynonenal (4HNE), rhodopsin, middle- and long-wavelength-sensitive opsin (M-opsin), short-wavelength-sensitive opsin (S-opsin), protein kinase Cα (PKCα) and glial fibrillary acid protein (GFAP). In addition, twelve-µm thick cryosections were prepared and examined to visualize the expression pattern of ionized calcium binding adaptor molecule 1 (Iba1) and CD68 in the retina. Detailed information on the primary and secondary antibodies used for IHC is listed in the Supplemental Table 1. Nuclear counterstaining was performed using DAPI. IHC images were acquired by the Leica LAS AF software under a fluorescent microscope (DM6000B, Leica, Germany). The camera gain and exposure setup for each indicated staining were kept the same across all experimental groups. The immunopositivity of the indicated immunostaining was analyzed using ImageJ. For quantification of the number of photoreceptor nuclei following HE staining, the multi-point tool in ImageJ was used to manually count the number of nuclei in the ONL on each macroscopic image (image size: 286.73 × 215.04 μm). For quantification of the 4HNE immunopositivity, the micrographic images (image size: 286.73 × 215.04 μm) were converted to 8-bit and the function of uncalibrated OD was applied under “calibrate”. Mean gray value and limit to threshold were then checked. After determining the target area using polygon tools, mean gray value was measured. Quantification of the fluorescence signals indicative of the immunopositivity was performed following the similar protocol as that for quantification of oxidized DHE, except that area and limit to threshold were checked under “set measurements”. The numbers from 4 images per section were averaged to generate the final number for each individual retina. Cautions were taken to ensure the sections were from the similar planes of the retina, the images were from the same region of the retina and the target area selection was kept consistent across different experimental groups.

### RNA Sequencing (RNA-seq)

The retinas were collected 1 d after the experimental light exposure. Total RNA was then isolated using a mirVana miRNA Isolation Kit (Thermo Fisher Scientific, USA) following the manufacturer’s protocols. After assessing the RNA integrity using an Agilent 2100 Bioanalyzer (Agilent Technologies, USA), the cDNA libraries were constructed using a TruSeq Stranded mRNA LT Sample Prep Kit (Illumina, San Diego, CA, USA). Libraries were then sequenced on an Illumina NovaSeq 6000 platform. Trimmomatic was used to process the raw data in the fastq format and the low-quality reads were removed. Hisat2 was adopted to map the clean reads to the reference genome. FPKM (fragments per kilobase of transcript per million fragments mapped) of each gene was calculated using cufflinks and the read counts of each gene were obtained by htseq-count. Principal component analysis (PCA) was performed to visualize the distribution and variation of the samples. Differentially expressed genes (DEGs) were identified using the *DESeq2* R Package functions estimateSizeFactors and nbinomTest. Statistical significance in the differentially expressed genes was defined as P value < 0.05 and fold change > 1.5. Hierarchical cluster analysis of DEGs was performed to visualize the expression pattern of DEGs from the indicated treatment groups. To perform functional enrichment analysis, the gene set enrichment analysis (GSEA) was performed based on Kyoto Encyclopedia of Genes and Genomes (KEGG) and Gene Ontology (GO) using *ClusterProfiler* R package and org.Mm.eg.db annotation package. The gene set with an absolute normalized enrichment score (NES) > 1, p value < 0.05 and false discovery rate (FDR)-adjusted p-value < 0.25 was considered to be significantly enriched. The Benjamini-Hochberg procedure was used to control the FDR.

### Real-Time Quantitative Polymerase Chain Reaction (qPCR)

The retinas were collected 1 d after the experimental light exposure. Total RNA was then isolated using TRIzol reagent (Invitrogen, USA), followed by reverse transcription using PrimeScript RT Master Mix (TaKaRa, Japan). The mRNA expression of the indicated genes was analyzed using the LightCycler 480 SYBR Green I Master (Roche, Germany) on a LightCycler 480 II system (Roche, USA). 18s rRNA was analyzed in parallel as an internal control. The primer sequences were included in Supplemental Table 2. The fold change in the candidate gene expression was calculated according to 2 ^−[Ct (candidate)−Ct (18s rRNA)]^.

### Statistical Analysis

The data were presented as mean ± standard error of mean (SEM). The Kolmogorov-Smirnov test and the Shapiro-Wilk test were used to test the normality of the data. The statistical analyses were then performed by Student’s *t*-test or one-way ANOVA with the Turkey multiple-comparisons test. Statistical significance was defined as P < 0.05.

## Results

### Re Treatment Attenuates Photooxidative Stress in the Retina

**Fig. 1:**
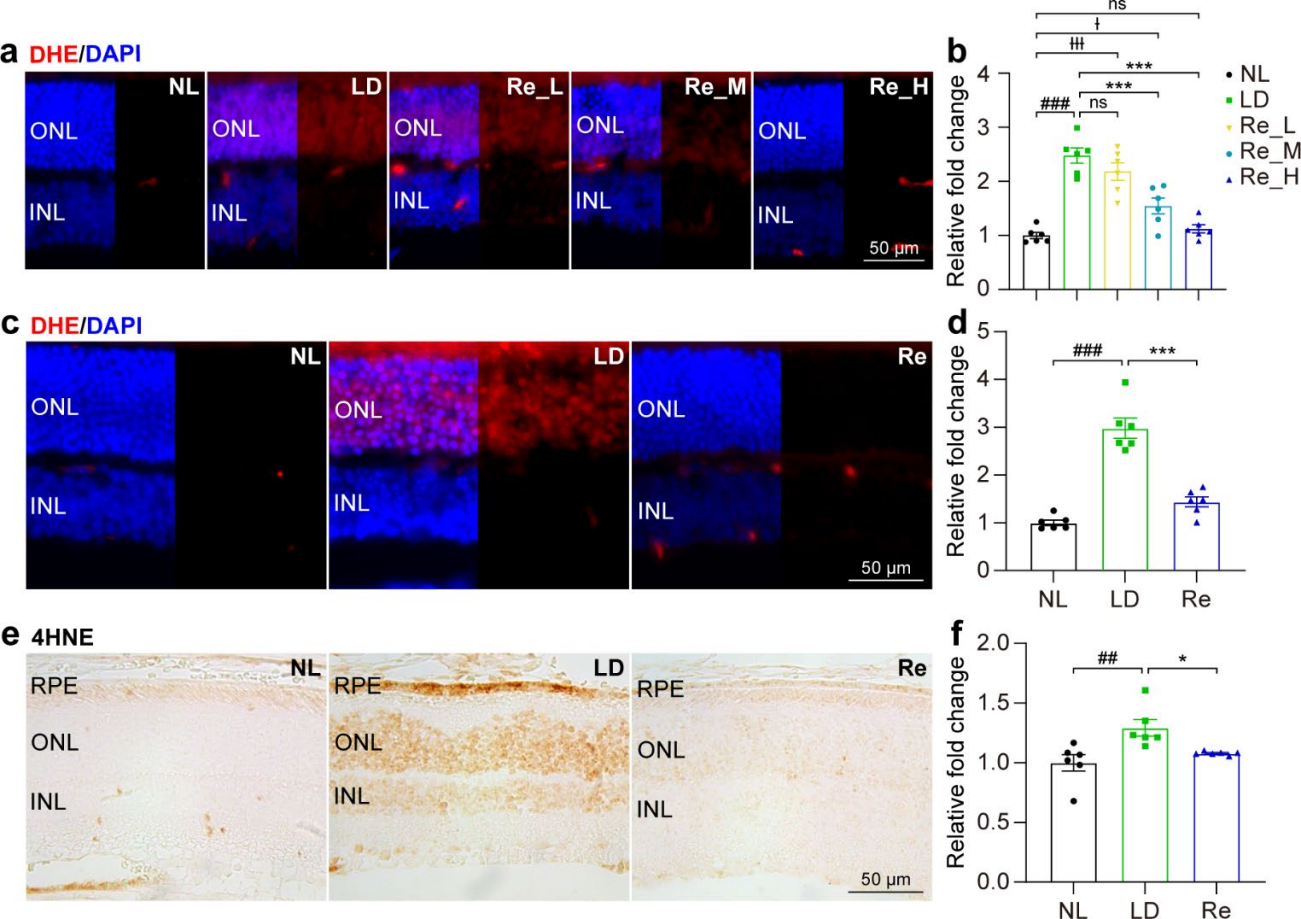
**Re-treatment attenuates ROS production and lipid peroxidation in the light-exposed retinas. (a)** Retinal ROS production (in red) 3 h after light exposure. DAPI positivity (in blue) highlighted nuclei. NL, the vehicle-treated mice unexposed to the experimental light exposure; LD, the light-exposed vehicle-treated mice; Re_L, the light-exposed mice treated with 5 mg/kg Re; Re_M, the light-exposed mice treated with 25 mg/kg Re; Re_H, the light-exposed mice treated with 100 mg/kg Re. Scale bar, 50 μm. **(b)** Quantification of ROS signal in the ONL. Relative fold change was plotted against NL. **(c)** Retinal ROS production (in red) 1d after light exposure. Re, the light-exposed mice treated with 100 mg/kg Re. Scale bar, 50 μm. **(d)** Quantification of ROS signal in the ONL. Relative fold change was plotted against NL. **(e)** IHC of 4HNE immunopositivity in the retina 1d after the light exposure. Scale bar, 50 μm. **(f)** Quantification of the immunopositivity of 4HNE in the ONL. Relative fold change was plotted against NL. Data were expressed as mean ± SEM (n = 6 per group). ^##^ Compared to NL, P < 0.01; ^###^ compared to NL, P < 0.001; * compared to LD, P < 0.05; *** compared to LD, P < 0.001; ^Ɨ^ compared to NL, p < 0.05; ^ƗƗƗ^ compared to NL, p < 0.001. ns, not significant. INL, inner nuclear layer; ONL, outer nuclear layer; RPE, retinal pigment epithelium

To assess the pharmacological impact of Re on photooxidative stress, the level of ROS was examined in the retina in situ 3 h after the experimental light exposure and the indicated treatments, which included Re treatment administered at 5, 25 and 100 mg/kg. As shown in the Fig. 1a and b, the ROS signal was barely detected in the outer nuclear layer (ONL) in the retinas from the normal controls; however, the level of ROS was evidently increased in the ONL in the vehicle-treated light-exposed retinas. On the other hand, reductions in the ROS signal in the ONL were readily detected in the light-exposed mice treated with Re at 25 and 100 mg/kg. Given that comparable levels of ROS signal in the ONL were noted in the normal controls and the light-exposed mice treated with 100 mg/kg Re, Re treatment was repeated at 100 mg/kg, followed by examination of the retinal ROS production 1 d after the experimental light exposure. As shown in Fig. 1c and d, the pronounced increase in the ROS signal was consistently observed in the ONL in the light-exposed vehicle-treated retinas compared to the normal controls. In sharp contrast, much less ROS signal was detected in the ONL in the light-exposed Re-treated retinas. Furthermore, given that the mechanisms of the anti-oxidant properties of Re implicate alleviating lipid peroxidation (Verstraeten et al. 2020) and lipid peroxidation is a major consequence of oxidative stress in photoreceptors, IHC was further performed to examine the immunopositivity of 4HNE, a marker for lipid peroxidation, in the retina. As shown in Fig. 1e and f, increased immunopositivity of 4HNE was readily detected in the ONL and retinal pigment epithelium (RPE) in the light-exposed vehicle-treated retinas compared to the normal controls. In contrast, significantly decreased 4HNE immunopositivity was noted in the light-exposed Re-treated retinas compared to the light-exposed vehicle-treated retinas. These results collectively indicate that Re is effective at attenuating photooxidative stress in the retina.

### Re Protects Against Photooxidative Stress-Mediated Impairment of the Photoreceptor Morphological Integrity

**Fig. 2:**
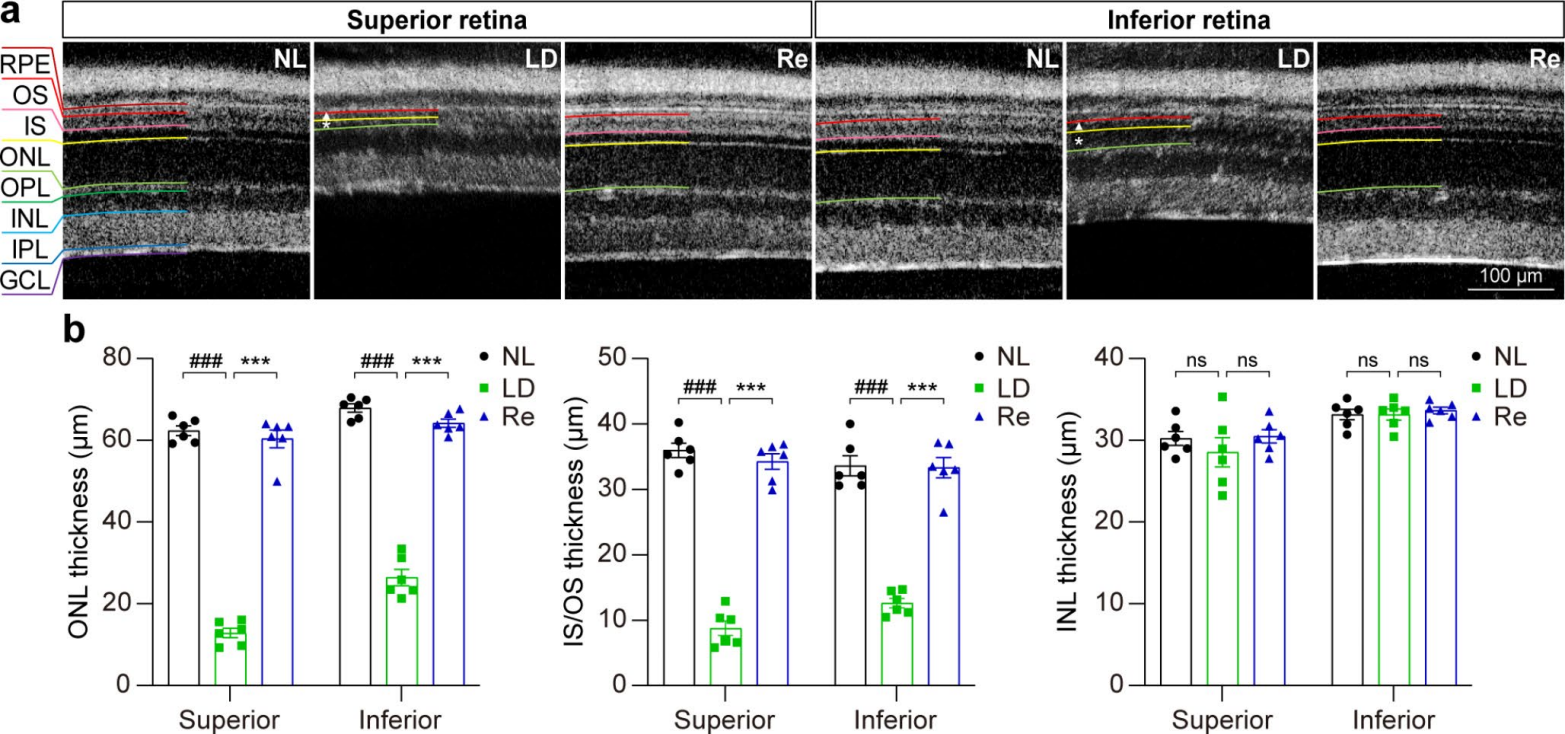
**Re-treatment attenuates morphological impairment of photoreceptors in the light-exposed retinas. (a)** OCT scans of the retina. NL, the vehicle-treated mice unexposed to the experimental light exposure; LD, the light-exposed vehicle-treated mice; Re, the light-exposed mice treated with 100 mg/kg Re. **(b)** The thickness of the ONL, IS/OS and INL at 500 μm off the ONH in the superior and inferior retina. White asterisk, impaired ONL. Closed triangle, IS/OS. Scale bar, 100 μm. Data were expressed as mean ± SEM (n = 6 per group). ^###^ Compared to NL, P < 0.001; *** compared to LD, P < 0.001; ns, not significant. GCL, ganglion cell layer; INL, inner nuclear layer; IPL, inner plexiform layer; IS, inner segment; ONH, optic nerve head; ONL, outer nuclear layer; OPL, outer plexiform layer; OS, outer segment; RPE, retinal pigment epithelium

As shown above, Re treatment attenuates light-induced photooxidative stress in the retina. Given that photooxidative stress is the key mechanism leading to photoreceptor degeneration, the potential protective effects of Re against photooxidative stress-mediated loss of photoreceptors were further assessed using full-retinal cross-sectional OCT scans after the indicated treatments. The experimental light exposure resulted in selective diminishment of the photoreceptor IS/OS and ONL in both superior and inferior retinas without causing overt morphological changes in the inner retina. In contrast, no significant changes in the photoreceptor morphological integrity were observed in the light-exposed Re-treated mice (Fig. 2a). Consistently, the ONL and IS/OS but not the inner nuclear layer (INL) was much thinner in the superior and inferior retinas in the light-exposed vehicle-treated retina compared to the normal controls, whereas the thickness of ONL and IS/OS was increased in the inferior and superior retinas in the light-exposed mice Re-treated mice (Fig. 2b). Furthermore, the protection of Re against photooxidative stress-mediated photoreceptor degeneration was confirmed following HE examination of the retinal gross morphology. Obliteration of the photoreceptor OS, IS and ONL was observed in the light-exposed vehicle-treated retinas; however, the morphological features of the photoreceptor OS, IS and ONL were to a large extent preserved in the light-exposed Re-treated mice (Supplemental Fig. 1a). Consistently, the number of photoreceptor nuclei was significantly decreased in the light-exposed vehicle-treated retinas. In sharp contrast, the number of photoreceptor nuclei was remarkably increased in the light-exposed Re-treated mice (Supplemental Fig. 1b). Therefore, the results from the OCT imaging and HE examination collectively demonstrate that Re protects against photooxidative stress-triggered photoreceptor morphological degeneration.

### Re Maintains the Retinal Function Under Photooxidative Stress Conditions

**Fig. 3:**
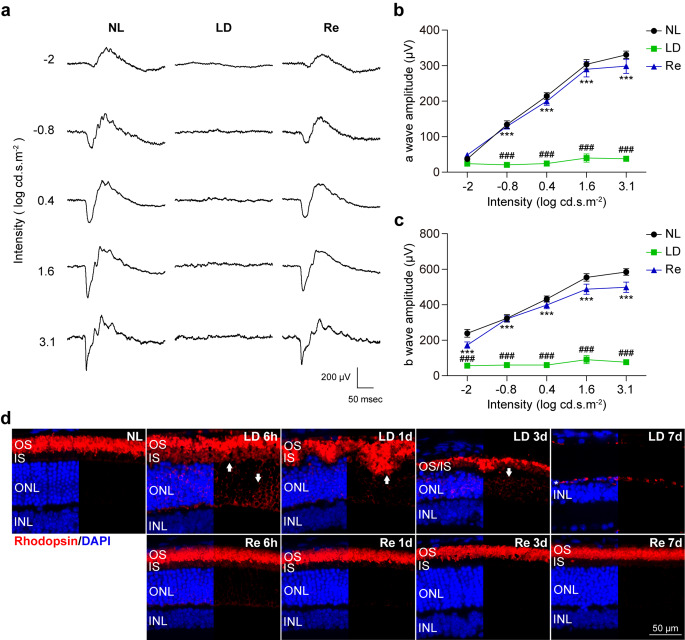
**Re maintains the retinal function and preserves the expression of rhodopsin in the light-exposed retinas. (a)** Representative electroretinograms from scotopic ERG recordings. **(b)** Amplitudes of a-wave. **(c)** Amplitudes of b-wave. **(d)** IHC of rhodopsin (in red) and DAPI positive nuclei (in blue). White asterisk, the diminished ONL. White arrows, mislocalized rhodopsin. Scale bar, 50 μm. Data were expressed as mean ± SEM (n = 6 per group). ^###^ Compared to NL, P < 0.001; *** compared to LD, P < 0.001. INL, inner nuclear layer; IS, inner segment; ONL, outer nuclear layer; OS, outer segment

Next, ERG was performed to further evaluate the pharmacological impact of Re on the retinal function under photooxidative stress conditions. In the retinas from the normal controls, the flash of light induced a typical biphasic waveform consisting of the first negative wave component, a-wave, and the following positive wave component, b-wave. In distinct contrast, this biphasic ERG waveform was evidently flattened in the light-exposed vehicle-treated retinas. However, the ERG responses from the light-exposed Re-treated retinas yielded biphasic ERG waveform patterns similar to the normal controls (Fig. 3a). Meanwhile, light stimuli induced intensity-dependent increases in the scotopic a-wave (Fig. 3b) and b-wave amplitudes (Fig. 3c) in the normal controls. Consistently, light intensity-dependent increases in the a-wave and b-wave amplitudes were markedly reduced in the light-exposed vehicle-treated retinas compared to the normal controls. The a-wave and b-wave amplitudes in the light-exposed Re-treated retinas were significantly increased compared to the light-exposed vehicle-treated retinas (Fig. 3b and c). Thus, the results from the ERG recording demonstrate that Re effectively maintains the retinal function under photooxidative stress conditions.

Well-maintained ERG responses prompted us to further probe the histopathological mechanisms of Re-conferred retinal functional protection under photooxidative stress conditions. As photoreceptors contribute to the a-wave response in ERG (Breton et al. 1994), IHC was performed to assess the impact of Re treatment on the morphology of rod and cone photoreceptors in situ. First, we examined the expression of signature proteins expressed in the photoreceptor OS, including rhodopsin, an essential functional protein of rod photoreceptors, S-opsin, a marker for the short-wavelength-sensitive cone photoreceptors and M-opsin, a marker for the middle- and long-wavelength-sensitive cone photoreceptors. As shown in Fig. 3d, abundant expression of rhodopsin was readily detected in the OS in the normal controls. In sharp contrast, the OS-specific expression pattern of rhodopsin was overtly disrupted in the light-exposed vehicle-treated retinas 6 h and 1 d after the light exposure. By 3 d post the experimental light exposure, the expression of rhodopsin was remarkably diminished in the light-exposed vehicle-treated retinas. By 7 d after the light exposure, the expression of rhodopsin was barely detected in the light-exposed vehicle-treated retinas. On the contrary, Re treatment maintained the normal expression pattern of rhodopsin in the light-exposed retinas. Similar observations were made with respect to the expression pattern of S-opsin (Supplemental Fig. 2a) and M-opsin (Supplemental Fig. 2b). The results here demonstrate that Re treatment leads to well-maintained photoreceptor OS morphology under photooxidative stress conditions.

ERG b-wave reflects the function of bipolar cells (Stockton and Slaughter 1989). Our previous studies have demonstrated that during the course of photooxidative stress-mediated photoreceptor degeneration, the second-order retinal neurons bipolar cells manifest morphological impairment, which may in part contribute to the functional deterioration of the retinas (Chen et al. 2016). Thus, the effect of Re treatment on the morphology of the bipolar cells was also examined to further characterize the retinal protective effects of Re. The bipolar cells can be visualized by the expression of PKCα in the retina. IHC examination revealed that the dendrites of bipolar cells were markedly impaired (Supplemental Fig. 3a) and the immunopositivity of PKCα in the outer plexiform layer (OPL) was significantly reduced (Supplemental Fig. 3b) in the light-exposed vehicle-treated retinas compared to the normal controls. In contrast, increased immunopositivity of PKCα in the OPL outlined well-preserved bipolar cell dendritic terminals in the light-exposed Re-treated retinas (Supplemental Fig. 3a and b). These results indicate that Re treatment protects against photoreceptor degeneration-associated impairment of the second-order bipolar cells under photooxidative stress conditions.

### Re Treatment Counteracts the Deleterious Impact of Photooxidative Stress on the Retinal Gene Expression

**Fig. 4:**
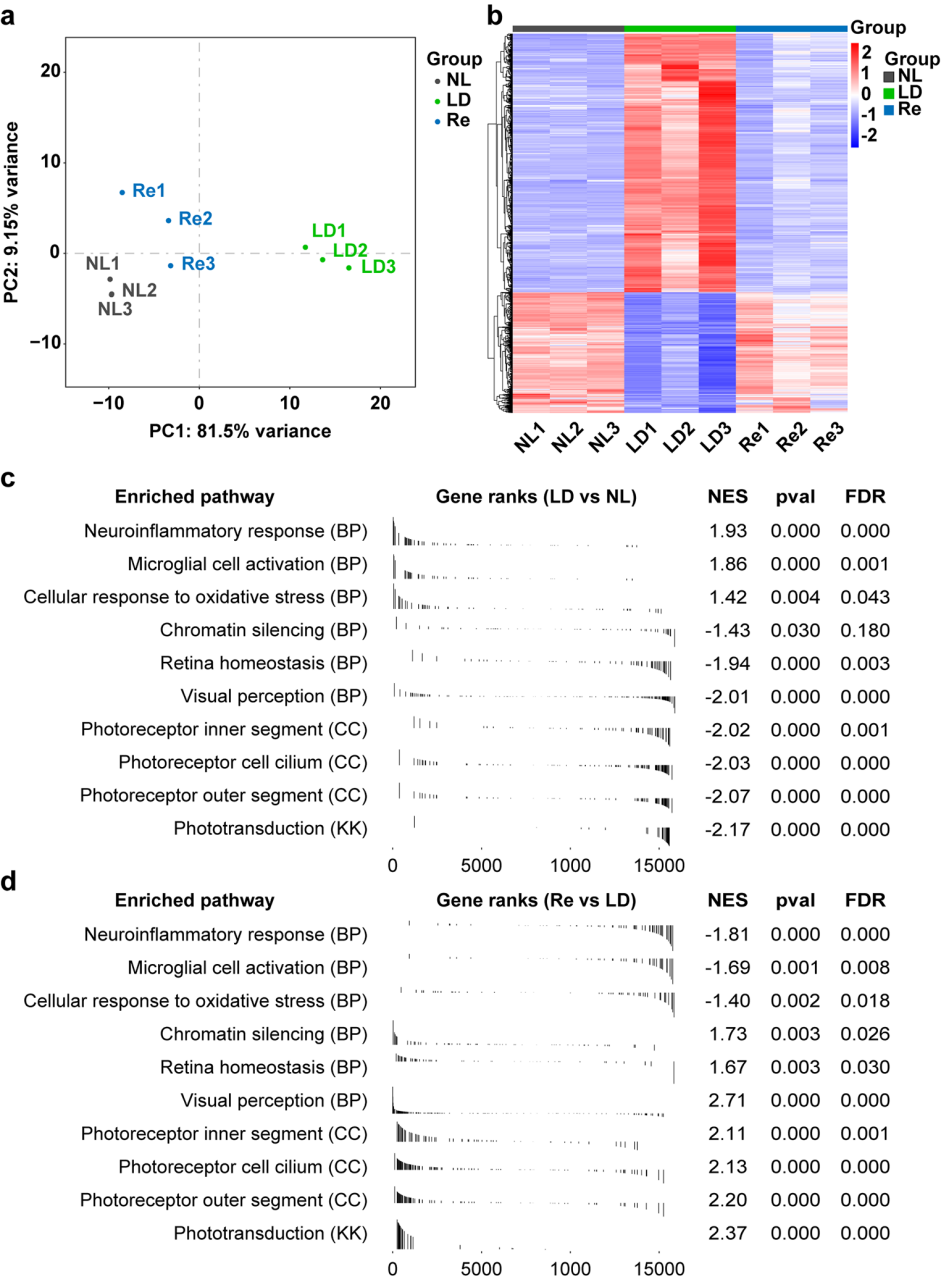
**Re-treatment counteracts light-induced alterations of the gene expression profiles in the retina. (a)** PCA of all data sets from NL (gray), LD (green) and Re (blue) (n = 3 per group). **(b)** Hierarchical clustering analysis of the DEGs from NL, LD and Re. Scale bar, the Z score indicative of upregulation (red) and downregulation (blue) of gene expression. **(c)** GSEA based on KEGG or GO gene sets (LD vs. NL). **(d)** GSEA based on KEGG or GO gene sets (Re vs. LD). FDR, false discovery rate; NES, normalized enrichment score; pval, p-value

Next, the retinas were collected at 1 d post experimental light exposure and subjected to RNA-seq analyses to further understand the mechanisms associated with the retinal protective effects of Re against photooxidative stress. Principal component analysis (PCA) provided an unbiased overview that the retinal gene expression profiles of the normal controls and that of the light-exposed vehicle-treated mice were overtly separated. However, the retinal gene expression profiles from the light-exposed Re-treated mice clustered closer to that from the normal controls (Fig. 4a). Consistently, hierarchical clustering analysis of the differentially expressed genes (DEGs) revealed that unlike the gene expression profiles from the light-exposed vehicle-treated retinas, the retinal gene expression profiles in the light-exposed Re-treated mice were similar to the normal controls (Fig. 4b). Additionally, gene set enrichment analysis (GSEA) of KEGG and GO revealed that experimental light exposure resulted in significant upregulation in the pathways such as neuroinflammatory response, microglial cell activation and cellular response to oxidative stress in the retina. On the other hand, pathways including chromatin silencing, retina homeostasis, visual perception, photoreceptor inner segment, photoreceptor cell cilium, photoreceptor outer segment and phototransduction were significantly downregulated in the light-exposed vehicle-treated retinas (Fig. 4c). However, compared to the light-exposed vehicle-treated retinas, the light-exposed Re-treated retinas were characterized by significantly downregulated pathways of neuroinflammatory response, microglial cell activation and cellular response to oxidative stress as well as upregulated pathways of chromatin silencing, retina homeostasis, visual perception, photoreceptor inner segment, photoreceptor cell cilium, photoreceptor outer segment and phototransduction (Fig. 4d). These results provide molecular evidence that further corroborates the retinal protective effects of Re against photooxidative stress-mediated photoreceptor degeneration.

**Fig. 5:**
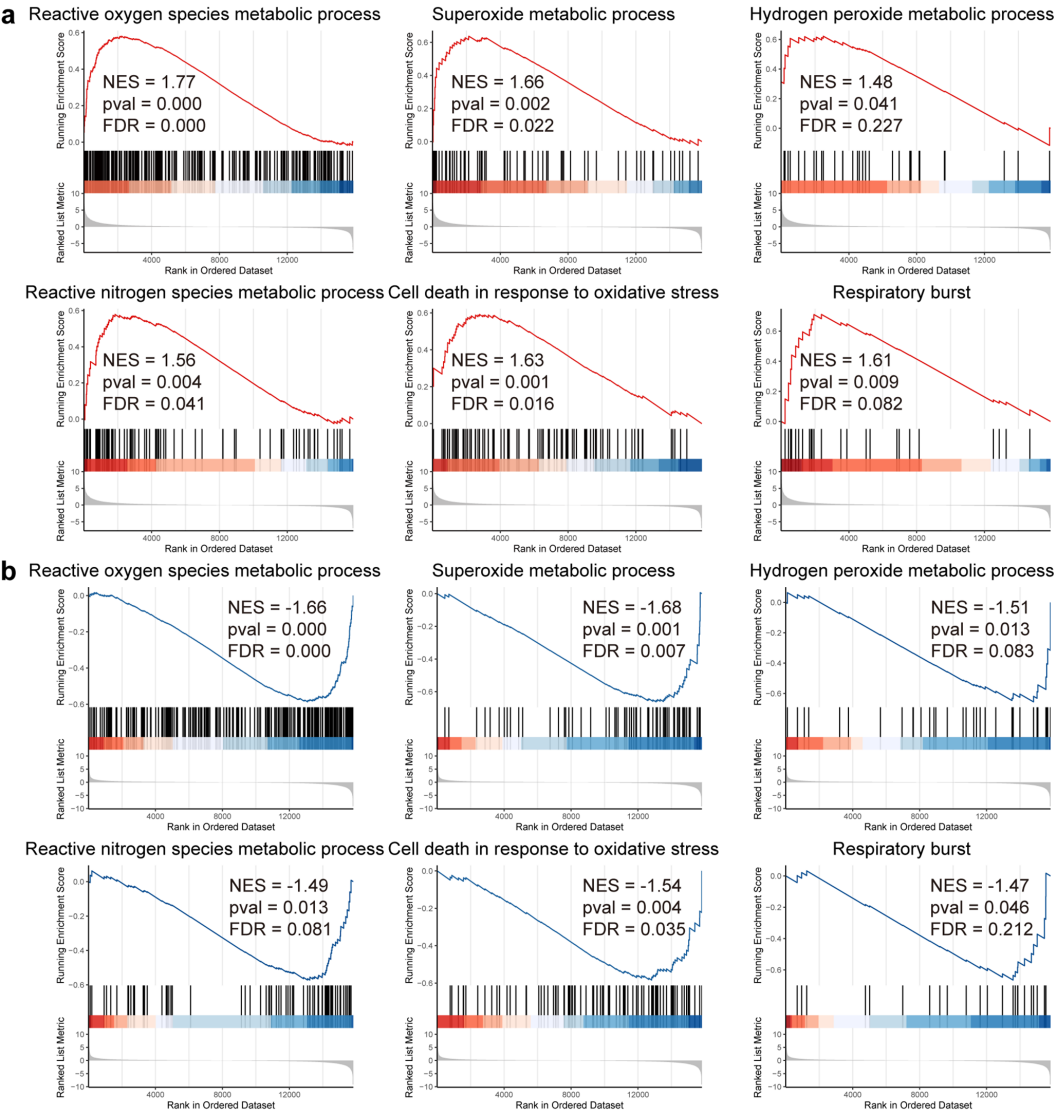
**Re treatment counteracts light-induced alterations of the gene sets associated with oxidative stress in the retina. (a)** Positively enriched oxidative stress-related gene sets in the light-exposed vehicle-treated retinas as revealed by GSEA. **(b)** Negatively enriched oxidative stress-related gene sets in the light-exposed Re-treated retinas as revealed by GSEA. FDR, false discovery rate; NES, normalized enrichment score; pval, p-value

A further analysis of the pathways associated with oxidative stress unveiled that gene sets such as reactive oxygen species metabolic process, superoxide metabolic process, hydrogen peroxide metabolic process, reactive nitrogen species metabolic process, cell death in response to oxidative stress and respiratory burst were significantly upregulated in the light-exposed vehicle-treated retinas (Fig. 5a). In contrast, these gene sets were noted to be significantly downregulated in the light-exposed Re-treated retinas compared to the light-exposed vehicle-treated retinas (Fig. 5b). Taken together, the results from the transcriptomic analyses provide a non-biased overview of the molecular pathways associated with Re-conferred retinal protection under photooxidative stress conditions.

### Re Treatment Counters Photooxidative Stress-Triggered Downregulation of Genes Implicated in Phototransduction

**Fig. 6:**
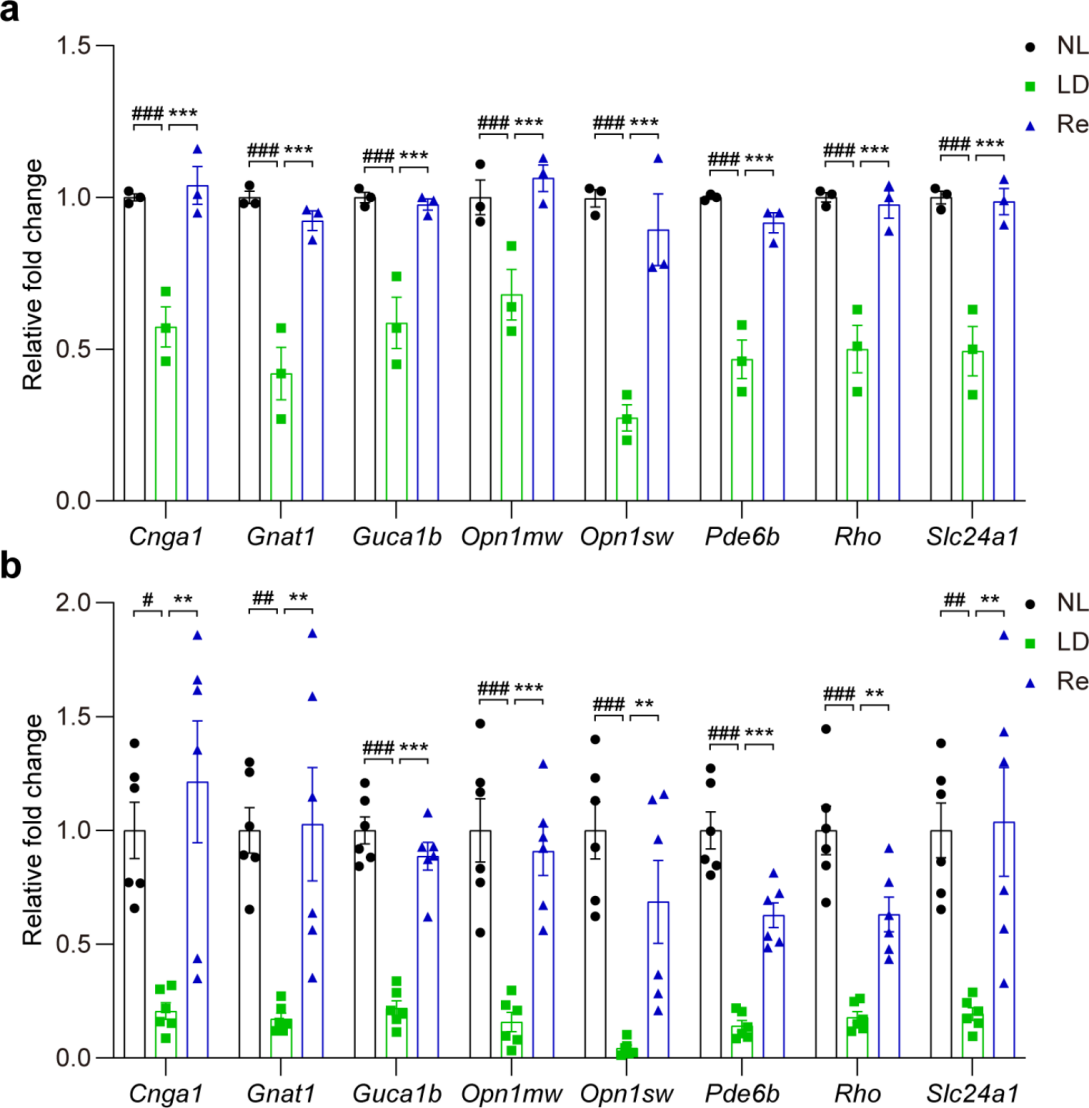
**Re maintains the retinal expression of genes essential for phototransduction in the light-exposed retinas. (a)** The retinal expression of phototransduction genes revealed by RNA-seq (n = 3 per group). **(b)** Real-time qPCR validation of the retina expression of phototransduction genes (n = 6 per group). Relative fold change was plotted against NL. Data were expressed as mean ± SEM. ^#^ Compared to NL, P < 0.05; ^##^ compared to NL, P < 0.01; ^###^ compared to NL, P < 0.001; ** compared to LD, P < 0.01; *** compared to that from LD, P < 0.001

Photoreceptors are specialized retinal neurons equipped with the function to convert light into electrical signals, a process defined as phototransduction. Phototransduction initiates visual processes and thereby is indispensable for vision formation (Mannu 2014). As shown above, Re treatment counteracted photooxidative stress-triggered downregulation of phototransduction gene set in the retina (Fig. 4d), corroborating the functional preservation conferred by Re treatment (Fig. 3a-c). Given the critical role of phototransduction in the retinal function, real-time qPCR was further performed to validate the impact of Re treatment on the retinal expression of phototransduction gene signatures revealed by RNA-seq analyses, including rod cyclic GMP-gated cation channel subunit encoding gene *Cnga1*, rod-specific Gα transducin subunit encoding gene *Gnat1*, guanylate cyclase activator protein GCAP1 encoding gene *Guca1b*, M-opsin encoding gene *Opn1mw*, S-opsin encoding gene *Opn1sw*, phosphodiesterase 6 encoding gene *Pde6b*, rhodopsin encoding gene *Rho*, and *Slc24a1*, which encodes a member of the potassium-dependent sodium/calcium exchanger protein family. Consistent with the results obtained from the RNA-seq analyses (Fig. 6a), real-time qPCR revealed significantly decreased retinal expression of *Cgna1*, *Gnat1*, *Guca1b*, *Opn1mw*, *Opn1sw*, *Pde6b*, *Rho*, and *Slc24a1* in the light-exposed vehicle-treated mice compared to the normal controls. In sharp contrast, significantly increased retinal expression of *Cgna1*, *Gnat1*, *Guca1b*, *Opn1mw*, *Opn1sw*, *Pde6b*, *Rho*, and *Slc24a1* was noted in the light-exposed Re-treated mice compared to the light-exposed vehicle-treated mice (Fig. 6b). These results confirm that Re treatment counteracts photooxidative stress-triggered downregulation of genes implicated in phototransduction.

### Re Attenuates Photoreceptor Degeneration-Associated Inflammation and Microglial Activation in the Retina

**Fig. 7:**
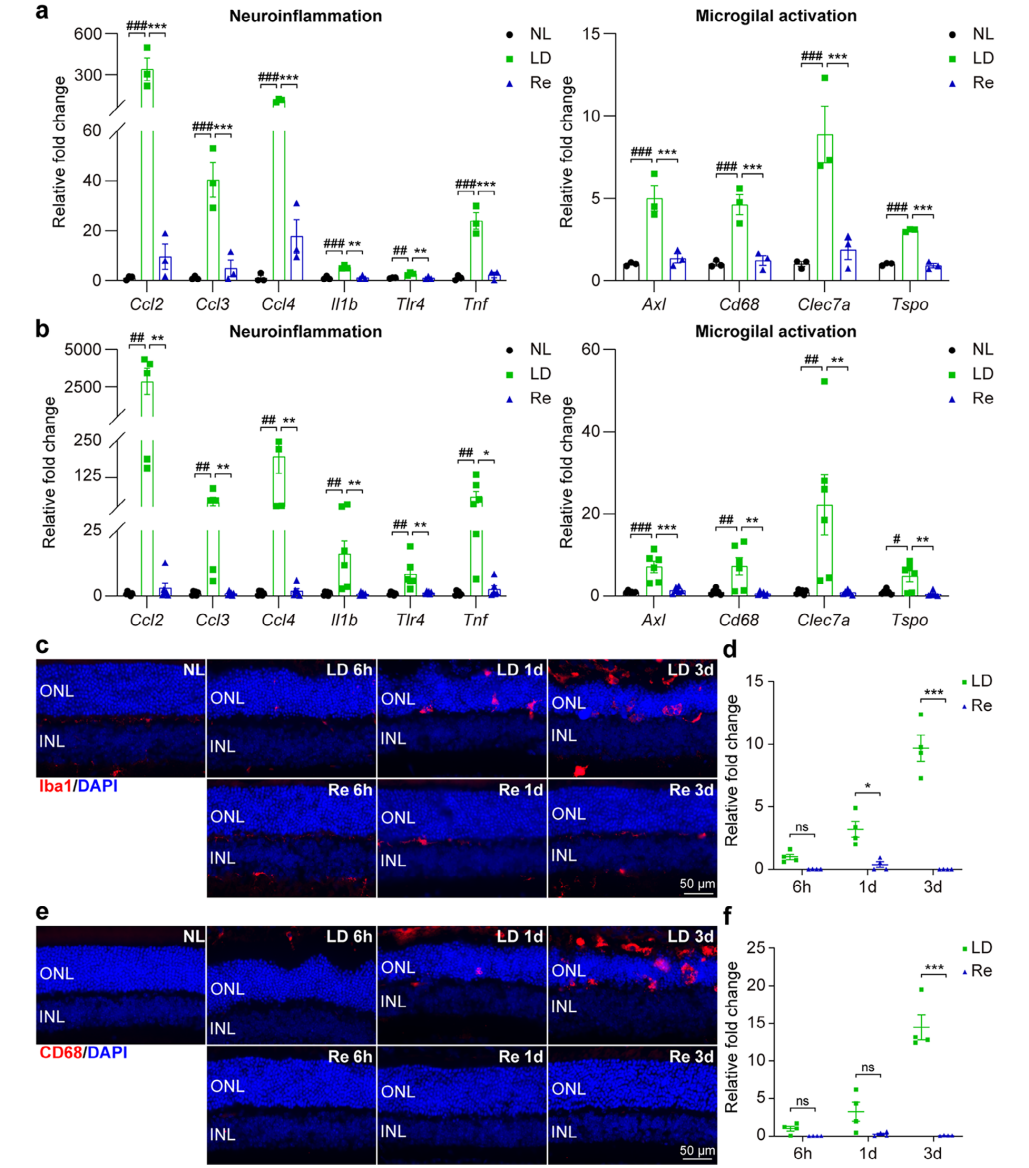
**Re treatment inhibits neuroinflammation and microglial activation in the light-exposed retinas. (a)** The retinal expression of the genes associated with neuroinflammation and microglial cell activation revealed by RNA-seq (n = 3 per group). Relative fold change was plotted against NL. **(b)** Real-time qPCR validation of the retinal expression of the genes associated with neuroinflammation and microglial cell activation (n = 6 per group). Relative fold change was plotted against NL. **(c)** IHC examination of Iba1 (in red) and DAPI positive nuclei (in blue). Scale bar, 50 μm. **(d)** Quantification of the Iba1 immunopositivity in the ONL and subretinal space. Relative fold change in the Iba1 immunopositivity was plotted against LD 6 h. **(e)** IHC examination of CD68 (in red) and DAPI positive nuclei (in blue). Scale bar, 50 μm. **(f)** Quantification of the CD68 immunopositivity in the ONL and subretinal space. Relative fold change in the CD68 immunopositivity was plotted against LD 6 h. Data were expressed as mean ± SEM. ^#^ Compared to NL, P < 0.05; ^##^ compared to NL, P < 0.01; ^###^ compared to NL, P < 0.001; * compared to LD, P < 0.05; ** compared to LD, P < 0.01; *** compared to LD, P < 0.001; ns, not significant

Based on the results from the RNA-seq analyses, neuroinflammatory response and microglial cell activation are the most upregulated pathways in the retina in response to the damaging light exposure (Fig. 4c). Re treatment resulted in a significant downregulation of gene sets of neuroinflammatory response and microglial cell activation in the retina (Fig. 4d). Mounting evidence has supported the pathophysiological significance of microglial activation and inflammation in the progression of photoreceptor degeneration (Rashid et al. 2019). Therefore, real-time qPCR was performed to validate the expression of neuroinflammatory genes including *Ccl2*, *Ccl3*, *Ccl4*, *Il1b*, *Tlr4* and *Tnf* as well as *Axl*, *Cd68*, *Clec7a* and *Tspo*, genes closely associated with microglial activation as revealed by the RNA-seq analyses (Fig. 7a). As shown in Fig. 7b, significantly elevated expression of *Ccl2*, *Ccl3*, *Ccl4*, *Il1b*, *Tlr4*, *Tnf*, *Axl*, *Cd68*, *Clec7a* and *Tspo* was observed in the light-exposed vehicle-treated retinas compared to the normal controls. In contrast, the retinal expression of these genes was significantly decreased in the light-exposed Re-treated mice compared to their light-exposed vehicle-treated counterparts. In addition, the immunopositivity of Iba1 and CD68 was also examined by IHC to visualize the changes of microglia in situ. The Iba1 positive cells were ectopically present in the ONL and the subretinal space in the light-exposed vehicle-treated mice 1 d and 3 d after the experimental light exposure. The ectopic localization of Iba1 positive cells in the ONL and the subretinal space was significantly attenuated in the light-exposed Re-treated retinas (Fig. 7c and d). Similar observations were made for the CD68 immunopositivity in the ONL and the subretinal space (Fig. 7e and f). Taken together, these results indicate that Re treatment suppresses microglial activation and neuroinflammatory responses in the light-exposed retinas.

### Re Preserves Müller Cell Homeostasis Under Photooxidative Stress Conditions

**Fig. 8:**
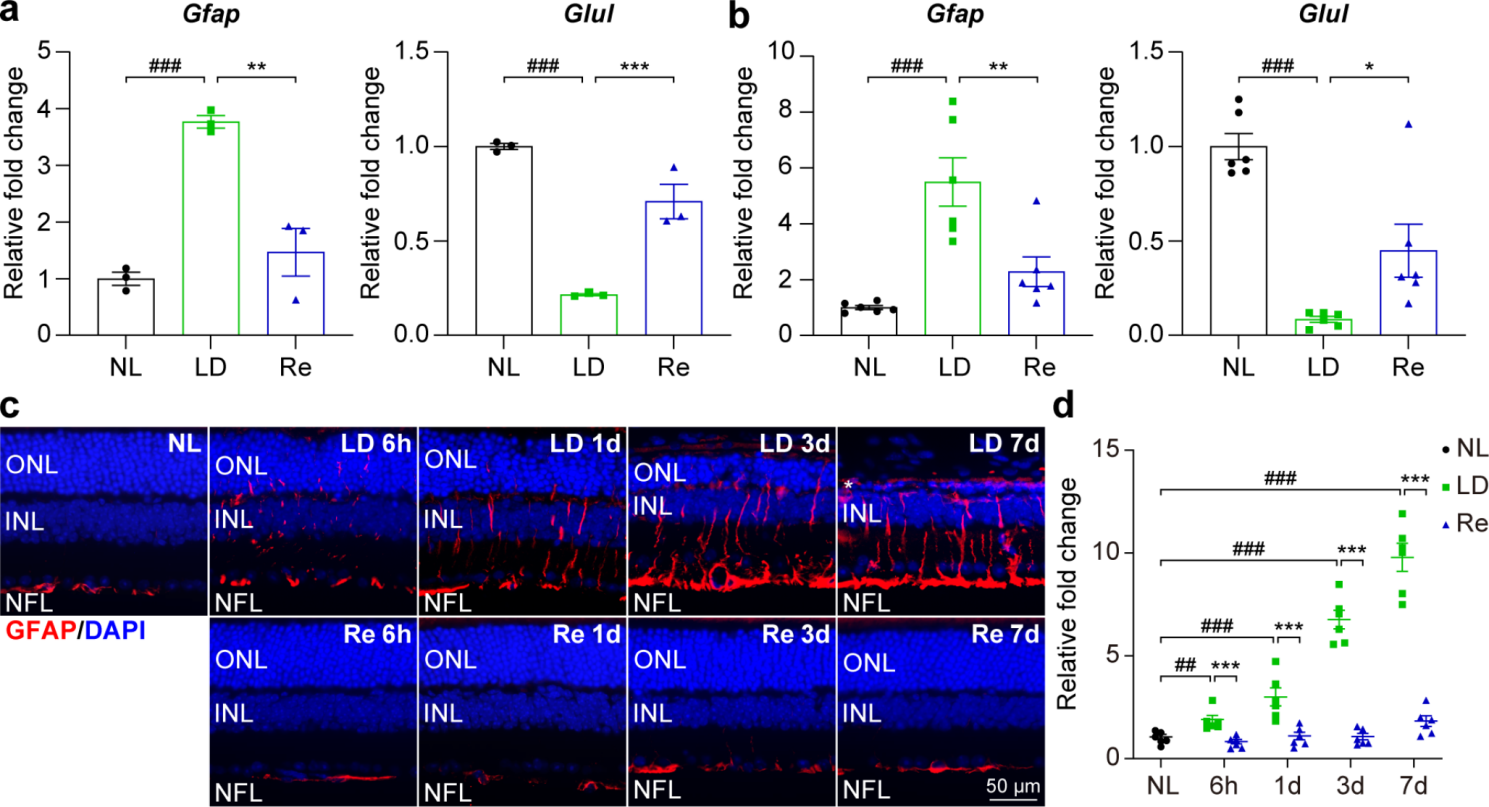
**Re treatment attenuates reactive gliosis of müller cells in the light-exposed retinas. (a)** The retina expression of *Gfap* and *Glul* revealed by RNA-seq (n = 3 per group). Relative fold change was plotted against NL. **(b)** Real-time qPCR verification of the retinal expression of *Gfap* and *Glul* (n = 6 per group). Relative fold change was plotted against NL. **(c)** IHC examination of GFAP (in red) and DAPI positive nuclei (in blue). Scale bar, 50 μm. **(d)** Quantification of the GFAP immunopositivity in the retina by ImageJ. Relative fold change in the GFAP immunopositivity was plotted against NL. Data were expressed as mean ± SEM. ^#^ Compared to NL, P < 0.05; ^##^ compared to NL, P < 0.01; ^###^ compared to NL, P < 0.001; * compared to LD, P < 0.05; ** compared to LD, P < 0.01; *** compared to LD, P < 0.001

In addition to the changes manifested by microglia during the course of photoreceptor degeneration, gliotic remodeling of the principal macroglia in the retina, müller cells, is encountered in nearly all types of retinal diseases including photoreceptor degeneration (Bringmann et al. 2006). Aside from causing functional impairment of the müller cells, reactive gliosis of müller cells may lead to the formation of the glial scar that in part contribute to deterioration of the retinal functional and structural integrity (Bringmann and Reichenbach 2001; Bringmann et al. 2006). Thus, the impact of Re treatment on reactive gliosis of müller cells was further assessed. First, a search into the data from the above-mentioned RNA-seq analyses revealed significantly increased retinal expression of *Gfap* in the light-exposed vehicle-treated retinas compared to the normal controls. Meanwhile, the expression of *Glul*, which encodes glutamine synthetase, a key enzyme that carries out the primary function of müller cells in glial-neuronal transmitter recycling, was significantly decreased in the light-exposed retinas. The upregulation of *Gfap* and concurrent downregulation of *Glul* support gliotic müller cells are functionally compromised under photooxidative stress conditions. In contrast, much lower expression of *Gfap* and significantly increased expression of *Glul* were noted in the light-exposed Re-treated retinas compared to the light-exposed vehicle-treated retinas (Fig. 8a). Moreover, validation of the retinal expression *Gfap* and *Glul* by real-time qPCR yielded similar results (Fig. 8b). In addition, the retinal expression pattern of Gfap was examined by IHC to directly visualize müller cell reactive gliosis in situ. As shown in Fig. 8c, the expression of Gfap was restricted to the nerve fiber layer (NFL) in the vehicle-treated normal controls. In distinct contrast, Gfap immunopositivity was noted to extend aberrantly across all layers of the retina in the light-exposed vehicle-treated retinas. Meanwhile, the aberrant pattern of Gfap was readily detected 6 h after the experimental light exposure and persisted 7 d after the light exposure. In sharp contrast to the light-exposed vehicle-treated retinas, the expression pattern of Gfap remained restricted to the NFL in the light-exposed Re-treated retinas (Fig. 8c). Compared to the normal controls, significantly increased Gfap immunopositivity across the retina was observed 6 h, 1 d, 3 d and 7 d after the light exposure in the vehicle-treated mice. The Gfap immunopositivity was much lower in the light-exposed Re-treated retinas at all the time points examined (Fig. 8d). These results collectively indicate that Re treatment alleviates müller cell reactive gliosis under photooxidative stress conditions.

## Discussion

Multiple lines of evidence presented in the current study support the pharmacological potentials of Re in attenuating photooxidative stress-mediated photoreceptor degeneration. The findings here demonstrate that Re is effective at inhibiting photooxidative stress and associated lipid peroxidation in the retina. Furthermore, Re treatment preserves the morphological and functional integrity of the retina under photooxidative stress conditions. Moreover, Re treatment counteracts the deleterious impact of photooxidative stress on retinal gene expression profiles. Lastly, Re protects against photoreceptor degeneration-associated reactive changes in microglia and müller cells.

Our findings here support novel pharmacological potentials of Re in mitigating retinal photooxidative stress. Photoreceptors are innately faced with the risk of heightened level of oxidative stress. Anatomically, photoreceptors are situated in an oxygen-rich environment due to close proximity to the choroidal vasculature that is featured with extremely high blood flow. Structurally, the photoreceptor OS is rich in peroxidation-susceptible polyunsaturated fatty acids. Metabolically, the photoreceptor IS is loaded with densely packed ROS-producing mitochondria to satisfy photoreceptors’ high level of energy demand. Functionally, photoreceptors are wired to absorb light and carry out phototransduction, during which photooxidative stress is naturally and unavoidably generated. Thus, equipped with the unique light-sensing functions, photoreceptors are naturally subjected to light damages, among which the photochemical damage caused by light exposure-generated ROS, namely photooxidative stress, is the most common form of the light damage. Photooxidative stress plays a pivotal role in the pathogenesis of photoreceptor degenerative disorders irrespective of etiologies (B. Domènech and Marfany 2020). Aside from causing oxidative damages to the macromolecules in the cell, photooxidative stress instigates oxidative damage of photoreceptor membrane lipids, leading to elevated level of lipid peroxidation. Lipid peroxidation spreads aggressively in a self-propagating chain reaction, resulting in exaggerated lipid peroxidation that amplifies the oxidative damage to photoreceptors (Tanito 2014). It has been demonstrated that Re is a major type of membrane-active ginsenosides with efficient antioxidant activities closely related to suppression of membrane lipid peroxidation. Suppressing membrane lipid peroxidation in part contribute to the protective effects of Re against ischemia/reperfusion-induced neuronal damage in the brain (Zhou et al. 2006; Verstraeten et al. 2020). In the current study, we further demonstrate that Re is effective at mitigating photooxidative stress in the retina (Fig. 1). The results from the whole-genome transcriptomic analyses corroborate the in situ histopathological findings of the suppressive effects of Re on the retinal photooxidative stress. Re treatment counteracts photooxidative stress-induced upregulation of multiple oxidative stress-related pathways and gene sets (Figs. 4 and 5), providing non-biased molecular evidence supporting the effect of Re at attenuating retinal photooxidative stress. The suppressive effects of Re on photooxidative stress in the retina help further expand the pharmacological understanding of the antioxidant properties of Re.

Most importantly, the current study demonstrates that the antioxidant activities of Re at attenuating photooxidative stress are translatable to the protection of photoreceptor structure and retinal function in vivo. Full-retinal OCT scans and histological examinations unveil that Re protects against photooxidative stress-mediated photoreceptor morphological degeneration and loss of photoreceptors (Fig. 2 and Supplemental Fig. 1). ERG recordings reveal that Re mitigates light-induced deterioration of the retinal function (Fig. 3a to c). These results collectively provide new understanding of the protective effects of Re on the photoreceptor structure and retinal function at the tissue level. Furthermore, histopathological findings also suggest that the photoreceptor-protective property of Re is associated with the effects at mitigating opsin mis-trafficking under photooxidative stress conditions. Prior to remarkable loss of rhodopsin expression, mislocalized rhodopsin in the photoreceptor IS and ONL could be observed shortly after the experimental light exposure (6 h and 1d), suggesting mis-trafficking of rhodopsin under photooxidative stress conditions (Fig. 3d). Similarly dysregulated expression patterns of S-opsin and M-opsin were observed (Supplemental Fig. 2). Rhodopsin is localized predominantly in the membrane compartment of rod photoreceptor OS. Mislocalization of rhodopsin in the photoreceptor IS, ONL and synaptic terminal has been noted in various photoreceptor degenerative processes (Bowes et al. 1988; Nir et al. 1989; Edward et al. 1993). High-fidelity and efficient trafficking and ensuing proper localization of opsin proteins within photoreceptors are tightly coupled to the survival and function of photoreceptors (Alfinito and Townes-Anderson 2002). In contrast to the mislocalized rhodopsin observed in the light-exposed vehicle-treated mice retinas, well-maintained localization of rhodopsin, S-opsin and M-opsin in the photoreceptor OS was identified in the Re-treated retinas (Fig. 3d and Supplemental Fig. 2). Furthermore, at the gene expression level, Re antagonizes light-induced downregulation of the pathways closely related to photoreceptor morphological and functional integrity, including retina homeostasis, visual perception, photoreceptor inner segment, photoreceptor cell cilium, photoreceptor outer segment and phototransduction (Figs. 4 and 6). These results indicate that Re provides a comprehensive protection to the molecular building blocks essential for the maintenance of photoreceptor morphological and functional integrity. Taken together, the findings here support the pharmacological value of antioxidant Re in attenuating photooxidative stress-mediated photoreceptor degeneration.

In addition, the impact of Re on photoreceptor degeneration-associated retinal inflammation and microglial activation is also worth noting. Microglia are normally located in the inner plexiform layer and outer plexiform layer, playing important roles in immune surveillance. In response to retinal insults, microglia can proliferate and migrate to the lesion site. Although the tightly controlled response of macroglia helps with the repair and restoration of the retina homeostasis, sustained and exaggerated microglial activation is not merely a result from the retinal damage, but also a source of neuroinflammation, which is neurotoxic and may further exacerbate photoreceptor degenerative pathologies (Gupta et al. 2003; Scholz et al. 2015; Zhao et al. 2015; Silverman and Wong 2018; Guadagni et al. 2019; Rashid et al. 2019; Rathnasamy et al. 2019). Here we present histopathological and molecular evidence confirming the notion that retinas challenged by photooxidative stress are marked by microglial activation and heightened neuroinflammatory responses. Most notably, Re treatment leads to much less microglial activation and significantly decreased level of inflammatory responses in the retina (Figs. 4 and 7). Although it has been demonstrated that Re is equipped with direct activities at suppressing microglial inflammatory activation in vitro (Madhi et al. 2021), given that microglial activation is a secondary event triggered by photooxidative stress-initiated photoreceptor degeneration, it is possible that attenuated photoreceptor degeneration in part contributes to reduced level of neuroinflammatory responses and microglial activation in the Re-treated retinas. Future investigations are necessary to further delineate if Re directly suppresses photoreceptor degeneration-associated microglial activation.

Lastly, our findings also highlight the beneficial impact of Re on retina homeostasis given that Re treatment dampens reactive gliosis in the müller cells under photooxidative stress conditions. Aside from microglial activation, aberrant reactive gliosis of the retinal macroglia, müller cells, also plays an important role in the deterioration of the retinal function and irreversible progression of photoreceptor degeneration (Bringmann and Reichenbach 2001; Bringmann et al. 2006). Müller cells are the only retina-specific glia and constitute the largest population of glia in the retina, providing homeostatic support for the structure, metabolism and function of the retinal neurons. Müller cells are morphologically aligned with different layers of the retinal neurons and are usually among the first to detect even subtle retinal damages, reacting in a rapid manner to any perturbations of the retina homeostasis. In the context of photoreceptor degeneration, müller cells undergo hypertrophic remodeling and functional impairment characterized by significantly upregulated expression of intermediate filament-encoding genes such as *Gfap* and decreased expression of glutamine synthetase-encoding *Glul*, respectively (Pfeiffer et al. 2020). The hallmark hypertrophic remodeling of müller glia as a result of increased intermediate filament synthesis leads to formation of a dense and rigid fibrotic seal that may cause further harm to the function and structure of retinal neurons. Meanwhile, impaired function of müller cells has detrimental impact on neurotransmission, leading to impaired function of the retina. Glutamine synthetase is predominantly expressed in müller cells and functions to convert glutamate, the most widely used neurotransmitter utilized by the retinal neurons such as photoreceptors, bipolar cells and ganglion cells, to glutamine, which is subsequently transported back to the retinal neurons as a precursor to glutamate. Müller cell-mediated glutamate metabolism is therefore critical for normal transmission of light signals to take place. Inhibition of glutamine synthetase results in diminished glutamate in retinal neurons, leading to rapid decline of the retinal function (Barnett et al. 2000). Consistently, photooxidative stress-mediated photoreceptor degeneration is marked by dysregulated retinal expression of *Gfap* and *Glul* as well as aberrant hypertrophic remodeling of müller glia (Fig. 8). In contrast, Re treatment leads to decreased expression of *Gfa*p, increased expression of *Glul* and attenuated hypertrophic remodeling of müller cells in the light-exposed retinas (Fig. 8), which may in part contribute to the protective effects of Re on retinal structural and functional homeostasis. Future studies are worth pursuing to investigate the direct impact of Re on müller cell reactive gliosis.

However, although the findings here support the pharmacological potentials of Re in attenuating photooxidative stress-mediated photoreceptor degeneration, the current study is to a certain extent limited by analyzing the effects of Re in female mice. Therefore, future studies validating the effects of Re in both female and male mice are required in order to determine if Re-conferred photoreceptor protection against photooxidative stress-mediated degeneration can be potentially generalized to the entire population.

In conclusion, the work demonstrates for the first time that as an antioxidant with anti-inflammatory activities, Re is pharmacologically effective at attenuating photooxidative stress-mediated photoreceptor degeneration and associated neuroinflammatory responses in vivo. In the meantime, Re maintains the morphological integrity of the second-order retinal neurons and preserves the cellular and molecular homeostasis of retinal glia. Thus, the major findings here provide novel in vivo evidence that supports the pharmacological potentials of Re in the therapeutic control of photoreceptor degenerative disorders.

### Electronic Supplementary Material

Below is the link to the electronic supplementary material.

Supplementary Material 1

## Data Availability

Data and material will be available upon request.
