# Supplementary material for: Ginsenoside Re Mitigates Photooxidative Stress-Mediated Photoreceptor Degeneration and Retinal Inflammation
Source: J Neuroimmune Pharmacol. 2023 Jun 16;18(3):397–412. doi: 10.1007/s11481-023-10073-y (PMC10577105; doi:10.1007/s11481-023-10073-y)
Supplement: Supplementary file 1 — Supplementary Material 1 [file 11481_2023_10073_MOESM1_ESM.docx]

**Supplementary information**

**Supplemental Table 1 Primary antibodies used for IHC examinations**

| Antibody name | Dilution | Manufacturer | Catalogue number |
| --- | --- | --- | --- |
| rabbit anti-CD68 | 1:500 | Abcam, USA | ab125212 |
| rabbit anti-GFAP | 1:500 | DAKO, USA | Z0334 |
| mouse anti-4HNE | 1:200 | JaICA, Japan | MHN-020P |
| rabbit anti-Iba1 | 1:500 | FUJIFILM Wako Pure Chemical Corporation, Japan | 019–19741 |
| rabbit anti-M-opsin | 1:100 | Millipore, USA | AB5405 |
| rabbit anti-PKCα | 1:5000 | Sigma-Aldrich, USA | P4334 |
| mouse anti-Rhodopsin | 1:1000 | Novus, USA | NBP2-25160 |
| rabbit anti-S-opsin | 1:100 | Millipore, USA | AB5407 |

**Supplemental Table 2** **Primer sequences used for real-time qPCR analyses**

| Gene name | Forward primer (5’-3’) | Reverse primer (5’-3’) |
| --- | --- | --- |
| *Axl* | ATGGCCGACATTGCCAGTG | CGGTAGTAATCCCCGTTGTAGA |
| *Ccl2* | AGCTGTAGTTTTTGTCACCAAGC | GTGCTGAAGACCTTAGGGCA |
| *Ccl3* | ACTGCCTGCTGCTTCTCCTACA | ATGACACCTGGCTGGGAGCAAA |
| *Ccl4* | ACCCTCCCACTTCCTGCTGTTT | CTGTCTGCCTCTTTTGGTCAGG |
| *Cd68* | GGCGGTGGAATACAATGTGTCC | AGCAGGTCAAGGTGAACAGCTG |
| *Clec7a* | GACTTCAGCACTCAAGACATCC | TTGTGTCGCCAAAATGCTAGG |
| *Cnga1* | CGAGCCATTTGTGCTGCTTA | TCATGGTTAGTTTAATATCTGCGCT |
| *Gfap* | CGGCCCTGAGAGAGATTCG | TGGCGGCGATAGTCGTTAG |
| *Glul* | GAGGAGAATGGTCTGAAGTGC | ACCGGCAGAAAAGTCGTTGA |
| *Gnat1* | CCCGACTACGATGGACCTAAC | TTGACGTTCTGTGTGTCGGT |
| *Guca1b* | CTGGACATTGTGGAGGCGAT | GACAGCTGGCCGTCTCCATT |
| *Il1b* | TGCCACCTTTTGACAGTGATG | AAGGTCCACGGGAAAGACAC |
| *Opn1mw* | ATGCCTTCCACCCTCTTGTG | CTGTCTTGGAGGTGCTGGAA |
| *Opn1sw* | TCATCTTCTGTTTCATCATTCCTCT | CTTTTGTGTCGTAGCAGACTCTT |
| *Pde6b* | TGGAGAACCGTAAGGACATCGC | TCCTCACAGTCAGCAGGCTCTT |
| *Rho* | CCTTTGTCATCTACATGTTCGTGGT | CTTCCTTCTCTGCCTTCTGAGTGGT |
| *Slc24a1* | GTCAAGGTCTGAAGGTTTGGG | TCTTTGGTCGGAGTAACCGC |
| *Tlr4* | AGCTTCTCCAATTTTTCAGAACTTC | TGAGAGGTGGTGTAAGCCATGC |
| *Tnf* | ACGTCGTAGCAAACCACCAA | GCAGCCTTGTCCCTTGAAGA |
| *Tspo* | GAGCCTACTTTGTACGTGGCGA | GCTCTTTCCAGACTATGTAGGAG |
| *18S rRNA* | GAGGTTCGAAGACGATCAGA | TCGCTCCACCAACTAAGAAC |

**Supplementary Figure 1**


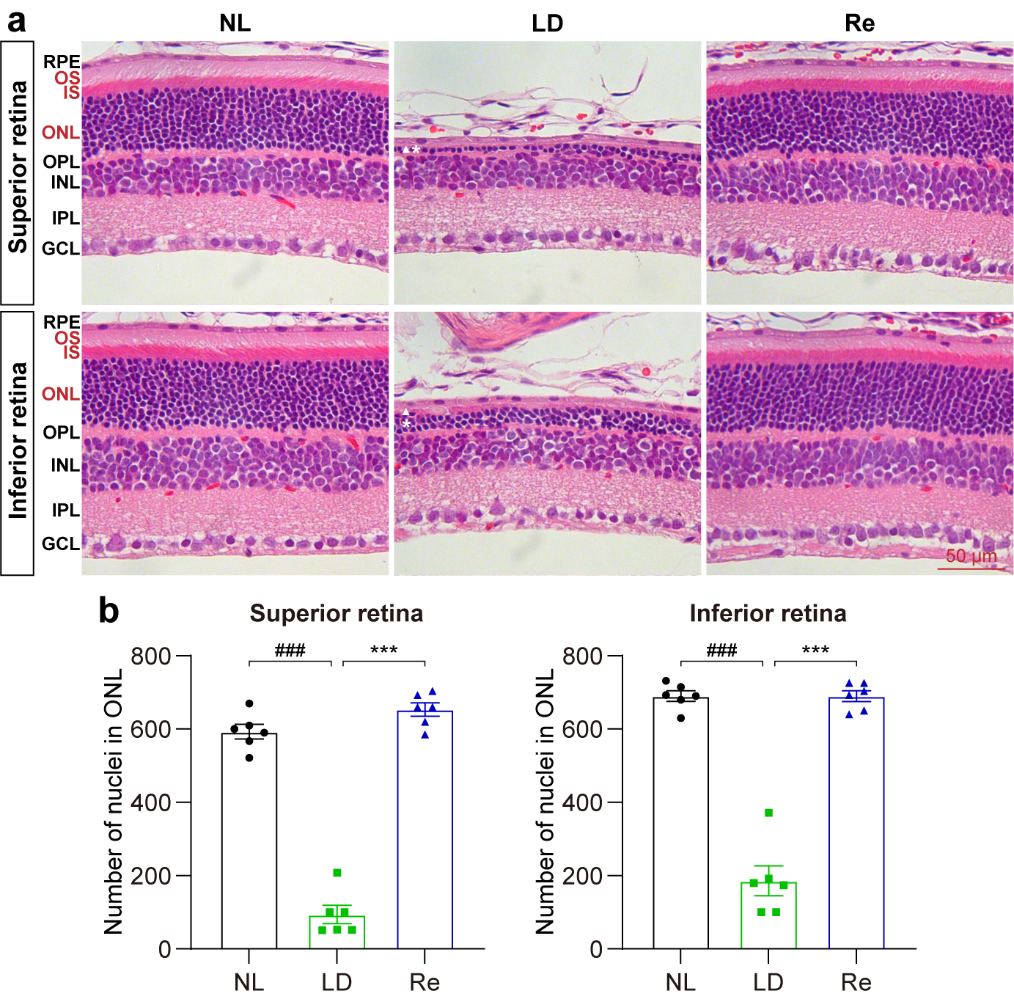


**Supplemental Fig. 1. Re treatment attenuates morphological impairment of photoreceptors in the light-exposed retinas. (a)** Histological examination of the retina. NL, the vehicle-treated mice unexposed to the experimental light exposure; LD, the light-exposed vehicle-treated mice; Re, the light-exposed mice treated with 100 mg/kg Re. Scale bar, 50 μm. **(b)** Quantification of the number of nuclei in the ONL. Data were expressed as mean±SEM (n=6 per group). ^###^ Compared to NL, P<0.001; *** compared to LD, P<0.001. GCL, ganglion cell layer; INL, inner nuclear layer; IPL, inner plexiform layer; IS, inner segment; ONL, outer nuclear layer; OPL, outer plexiform layer; OS, outer segment; RPE, retinal pigment epithelium.

**Supplementary Figure 2**


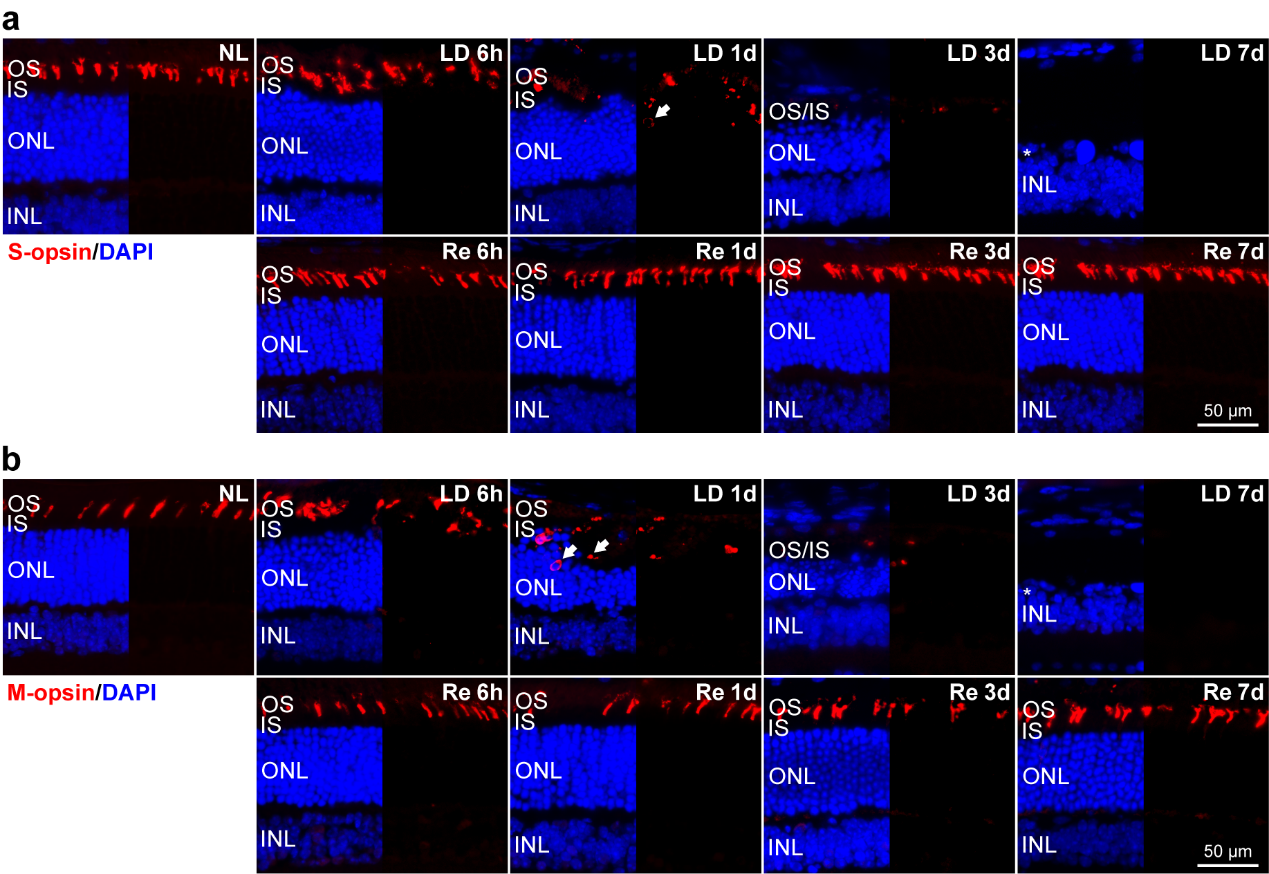


**Supplemental Fig.** **2. Re treatment** **preserves the expression of S-opsin and M-opsin in the light-exposed retinas.** **(a)** IHC of S-opsin (in red) and DAPI positive nuclei (in blue). White asterisk, the diminished ONL. White arrows, mislocalized S-opsin. Scale bar, 50 μm. **(b)** IHC of M-opsin (in red) and DAPI positive nuclei (in blue). White asterisk, the diminished ONL. White arrows, mislocalized M-opsin. Scale bar, 50 μm. INL, inner nuclear layer; IS, inner segment; ONL, outer nuclear layer; OS, outer segment.

**Supplementary Figure 3**


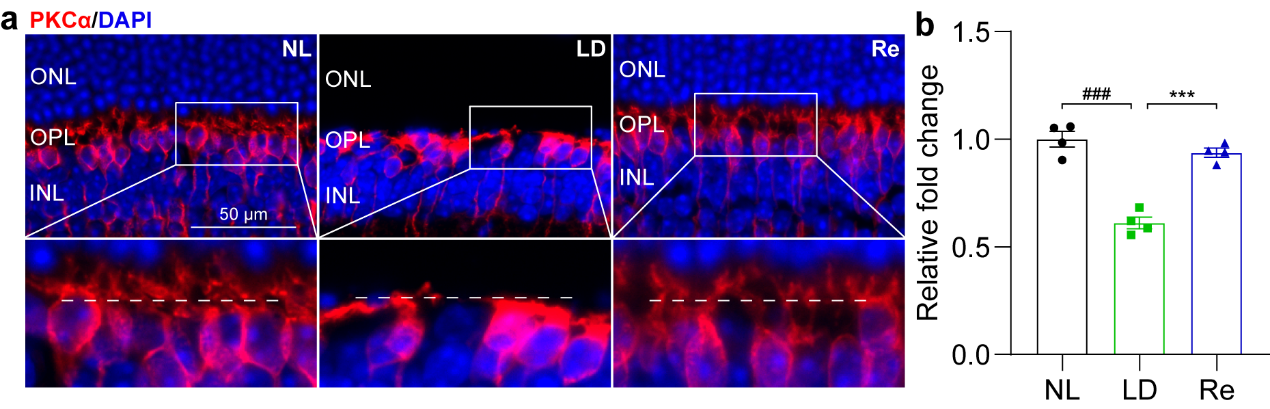


**Supplemental Fig. 3. Re treatment attenuates morphological impairment of bipolar cells in the light-exposed retinas. (a)** IHC of PKCα (in red) and DAPI positive nuclei (in blue). Scale bar, 50 μm. **(b)** Quantification of the PKCα immunopositivity in the OPL. Relative fold change in the PKCα immunopositivity in the OPL was plotted against NL. Data were expressed as mean±SEM (n=4 per group). ^###^ Compared to NL, P<0.001; *** compared to LD, P<0.001. INL, inner nuclear layer; ONL, outer nuclear layer; OPL, outer plexiform layer.
